# Case of Self-Mutilation

**Published:** 1868-07-01

**Authors:** Curtis T. Fenn

**Affiliations:** 234 Thirty-first street; Chicago


### CASE OF SELF-MUTILATION.

BY CURTIS T. FENN, M.D., CHICAGO.

G. S., aged 25, born in New York, a farmer, was admitted to the County Hospital, January 25th, 1868, in a condition of exhaustion. A portion of his clothing was saturated with blood. Examination showed that he had been castrated recently. His wounds were filled with fresh coagulum; and adhering to the clots of blood in his pantaloons, were two rude clamps of green sapling, tied with waxed end. He refused to answer any questions regarding himself; and it was, therefore, surmised that some rural outrage had been thus unmercifully revenged, at the hands of unknown swains. The natural simplicity and innocence of the man, made this appear doubtful, however. At length, he acknowledged to have committed the act himself; and gave the following history:

He had been inclined to solitude from childhood, and, at times, addicted to self-abuse. He was generally impressed with the idea that he should not be able to rid himself effectually of the habit, till he was castrated. He became afflicted with spermatorrhœa; and, although he felt well and strong, applied to the doctors for a cure, to no purpose. At length, one agreed to perform the operation, at his own request; but required a fee to begin with, which was more money than he could pay. With sublime hardihood, he now resolved to castrate himself, and so to obtain a deliverance which he considered essential to his future happiness. He ground two quadrilateral bits of iron to a cutting edge, and inserted them, an inch apart, into a block of wood. This instrument, he intended for making two separate parallel incisions at once, each to be two inches long. After a month's preparation, all was ready. His procedure then was as follows: For better concealment, and as like to receive more care when he should need it, he came to the city. The morning after his arrival, he seated himself astride an old trunk, in the upper story of his hotel, and contrived to spread his scrotum upon a surface of wood, placed between his thighs, and fasten it there. Taking the implement in one hand, he cautiously and deliberately rested its edges upon the stretched integument, and struck down upon it with a large stone, held in the other; thereby severing all the coverings of both testicles. He then dissected out the cords, and placed upon them rude clamps, he had made the day before. This part of the operation seems to have been performed with less resolution. However, he drew them as tightly together as he could, and, with one stroke of his jack-knife, cut off the offending members. In this condition, he set out for the County Hospital. On getting off the street-car at Eighteenth street, he fainted, and fell to the ground. The police had him taken up, and brought the rest of the way in an express wagon. He was immediately admitted, as an urgency case, and put to bed. The wounds healed by granulation. He expressed no regret, except at the danger of his exposure. He was discharged, cured, Feb. 17th, 1868.

234 *Thirty-first street*, *May* 23, 1868.

